# Where Should Papers Be Published?

**Published:** 1891-12

**Authors:** 


### Editorial.

### WHERE SHOULD PAPERS BE PUBLISHED?

The impression has gone abroad in the dental profession that, while it is numerically strong, it is lamentably lacking in intellectual and financial strength, and needs the help of the large business interests to enable it to advance. The time has come when dentists should not only be inspired with other views, but should refrain from all expressions that seem to lack faith, not in its ultimate possibilities, but in the position already attained.

We were impressed with the remarks made at the New York Odontological Society in June last, and published in the October number of this journal, and especially with those made by one gentleman present. The statement exhibited a condition of mental antagonism not at all creditable to the individual quoted by the speaker. In order that the subject may be understood, we give a portion of Dr. Bogue's remarks in reply to Dr. Meriam. After some preliminaries he stated:

"In making application as one of the members of your executive committee to a gentleman for a paper to be read before this Society, I was met with almost a prompt refusal, on the ground that papers read here would not be published in the *Dental Cosmos.* I undertook to argue the gentleman out of his decision, but was met by the *strong argument* that the *Dental Cosmos* had attained a position that made it the most *widely read of all the dental journals*; if he had taken the trouble to get up a good article he wished it to be read as widely as possible. . . . Now comes the question, Are we sufficiently strong to fight our own battles? Are we yet able to take the position as an independent profession, . . . casting aside personal self-interests and laboring for the good of others?" [Italics ours.]

The reasoning that would lead to the refusal of a paper on such grounds must be very defective. It is unquestionably true that to publish an article in certain dental periodicals, intended solely for advertisement, would be destructive, and result in burying the paper beyond all hopes of resurrection.

The question to be considered is, Have we in dentistry a large body of readers? Dentistry is physically a very trying profession, perhaps more so than any other. Very few persons after standing all day over the chair, subjected to the nervous anxieties of patients, are equal to spending their evenings in reading, much less in study. Besides this, it is well known that full practice means constant extra hours of labor, oftentimes far into the night, to keep up with the increasing demands of the clientele. This overtaxing work inevitably decreases the number of readers, and has also had the effect of limiting the number of those willing to write for societies and journals.

This being the fact, and it is indisputable, it naturally follows that those who will care to take professional journals must be confined to three classes,—

1. Those who desire to keep themselves fully abreast with the progress of scientific development, and who will endeavor to read everything as it appears, not only in one, but in several journals.

2. Those who take a journal to skim over its contents for any practical ideas, and give the advertising pages special attention for the same reason.

3. Those who take one journal solely for the advertisements.

It is probably true that of the fifteen thousand dentists in the United States, not more than forty per cent., or six thousand, judging by subscription-lists, take any journal regularly. Exactly how many may be classed under each of the three arbitrary divisions made it would be difficult to determine; but it is very safe to assume that one-half of the six thousand belong to the third class, who take a journal for its advertisements, while the balance may again be equally divided between the readers and those who seek only for practical ideas. This calculation reduces the leading journals to a common basis. Those which meet the higher wants of the first class will be taken and read, and it is immaterial whether the circulation be much or little, the result is the same as far as the distribution of ideas is concerned. If any doubt these conclusions, take any twenty dentists and interrogate them as to the contents of certain journals. The result, we think, will amply justify the statement made that dentists are not, regarded as a whole, devoted readers of their own literature.

If these conclusions be admitted, the person who habitually gives his productions to the journal supposed to have the largest circulation may not be making a mistake in so doing, if he wishes to have his article spread over the greatest number of pages; but he errs most decidedly if he supposes he will secure an increased number of readers.

That journal is the best and the surest medium for original thought which bases its reputation on the highest standard attainable, and which meets the needs of the most cultivated class in the profession.

The labor to sustain such journals is one worthy of our highest endeavor. It must be above selfish motives. It must recognize the fact that in such periodicals nothing is lost. They are the ones preserved, and eventually they become centres of reference. Libraries gladly receive them, and they develop in the largest sense not only the instructors of the present generation, but those which are to follow.

The remarks made at that meeting were unintentionally, we are disposed to believe, aimed at this journal. To disparage such an effort as the INTERNATIONAL DENTAL JOURNAL represents by bringing it in comparison with a journal of supposed larger circulation, may be interesting in a discussion, but is not the way, as we view it, to enlarge the sphere of professional usefulness. We are interested most in the fact that the journal alluded to, the INTERNATIONAL DENTAL JOURNAL, and all others of the character described, will be read equally, and their articles will be copied in proportion to their intrinsic value. In proof of this it may be stated that many of the papers published in this journal have been extensively republished abroad, and the subject-matter of several has elicited wide discussion. This has enlarged the circle of readers of some of the most valuable scientific articles published in the present volume.

The battle then can be, *must be,* fought within our own lines. Without vigorous and persistent effort no advance can be made. Opposition and contest means increased strength, and without it all effort becomes, in the end, weak and vacillating.

